# Transcriptomic and proteomic insights into feather keratin degradation by *Fervidobacterium*

**DOI:** 10.3389/fmicb.2025.1509937

**Published:** 2025-04-16

**Authors:** Rubén Javier-López, Mélodie Kielbasa, Jean Armengaud, Nils-Kåre Birkeland

**Affiliations:** ^1^Department of Biological Sciences, University of Bergen, Bergen, Norway; ^2^Département Médicaments et Technologies pour la Santé (DMTS), Université Paris Saclay, CEA, INRAE, Bagnols-sur-Cèze, France

**Keywords:** chicken feather, keratin, keratinase, oxidoreductase, peptidase, protease, proteomics, transcriptomics

## Abstract

Keratin, one of the most recalcitrant and abundant proteins on Earth, constitutes a challenging and underutilized material for the poultry industry. Although it resists degradation by most commonly available enzymes, natural breakdown occurs through the action of certain fungi and bacteria. This process remains poorly understood, and only a few thermophilic and anaerobic bacteria are known to effectively degrade keratin. Some members of the genus *Fervidobacterium* have been demonstrated to be effective at degrading feather keratin under high temperatures and anoxic conditions. However, a comprehensive evaluation of their keratinolytic capabilities remains lacking, leaving their potential largely underexplored. In this study, we assessed the keratinolytic activity of all available *Fervidobacterium* strains. Six strains were active against this recalcitrant substrate, namely *Fervidobacterium changbaicum* CBS-1^T^, *Fervidobacterium islandicum* H-21^T^, *Fervidobacterium pennivorans* T, *Fervidobacterium pennivorans* DSM9078^T^, *Fervidobacterium* sp. GSH, and *Fervidobacterium* sp. 21710. These bacteria were used in a comparative proteomics analysis, grown with either glucose or chicken feathers as the sole carbon source. Similarly, the three most efficient strains, *Fervidobacterium pennivorans* T, *Fervidobacterium* sp. GSH, and *Fervidobacterium islandicum* H-21^T^ underwent an in-depth comparative transcriptomics analysis. Among the numerous upregulated proteins and overexpressed genes identified when comparing feather-grown to glucose–grown cells, oxidoreductases and peptidases are key enzymes in the degradation process, suggesting their potential application in enzymatic keratinolytic cocktails for degrading feather keratin.

## Introduction

Keratin, a structural protein found in the epidermis and outer protective layers of vertebrates, provides protection, insulation, and other mechanical functions. Beta-keratin, one of the main forms of keratin, is the principal component of several structures, such as scales, beaks, and feathers (Zhang and Fan, 2021). Chicken feather keratin is rich in cysteine, glutamine, proline, and serine, and its composition is similar to that of other feather keratins (Saravanan and Dhurai, 2012). Thus, keratin is one of the most abundant proteins on Earth, with millions of tons of feathers produced annually as a by-product of the food industry (Shavandi et al., 2017; Chen et al., 2022), indicating both its prevalence and persistence in the environment. Characterized by a high cysteine content, typically ranging from 7 to 13%, the structure of keratin is strengthened by hydrogen and disulfide bonds, making it chemically stable (Lange et al., 2016; Shavandi et al., 2017) and resistant to most conventional hydrolytic enzymes commonly used for protein degradation (Sypka et al., 2021; Chen et al., 2022).

Considered a biowaste, feathers are a major concern for the food industry and constitute an underutilized residue that is traditionally transformed into low-value products, such as feather meal or fertilizers (De Oliveira Martinez et al., 2020), or even burned or discarded (Sahoo et al., 2017; Chen et al., 2022). Conventional keratin extraction methods typically disrupt the structure of wool or feathers, leading to alterations in the composition and generation of pollutants (Shavandi et al., 2017). Although keratin degradation occurs in nature, this reaction is slow, and the details of the degradation process are still to be fully elucidated at molecular level (Daroit and Brandelli, 2014; Sahoo et al., 2017). Keratin degradation has been hypothesized to involve the cleavage of disulfide bonds by oxidoreductases, followed by the combined action of endo-and exo-proteases and other enzymes. However, the details of the process at molecular level remain unknown (Lange et al., 2016; Shavandi et al., 2017; Qiu et al., 2020).

Certain groups of microorganisms, most of which are mesophilic aerobes, have been reported to break down feather keratin (Daroit and Brandelli, 2014; Sahni et al., 2015; Srivastava et al., 2020). Interestingly, only a few are anaerobic and thermophilic or hyperthermophilic, such as strains of the genus *Fervidobacterium,* belonging to the *Thermotogota* phylum. All members of this taxon have an external sheath-like membrane called the toga, which is a defining characteristic of this phylum (Huber et al., 1986; Bhandari and Gupta, 2014). All of them are thermophilic or hyperthermophilic and fermentative rods with optimal temperatures range of 65–80°C (Huber et al., 1990; Andrews and Patel, 1996; Friedrich and Antranikian, 1996; Cai et al., 2007; Podosokorskaya et al., 2011; Kanoksilapatham et al., 2016) that use various sugars and proteinaceous substrates as carbon and energy sources (Conners et al., 2006). Several strains of the *Fervidobacterium* genus can degrade feather keratin at high temperatures under anaerobic conditions, meaning that their enzymatic machinery is accordingly adapted to function in these conditions, and highlighting their biological and biotechnological relevance (Huber et al., 1990; Friedrich and Antranikian, 1996; Kanoksilapatham et al., 2016; Javier-Lopez et al., 2022; Wang et al., 2024). Moreover, degradation of recalcitrant compounds is more efficient at high temperatures, what makes thermophiles more attractive compared to their mesophilic counterparts (Cowan et al., 2024), explaining why thermophilic enzymes are widely used in industry (Atif et al., 2024). Their genomes are approximately two megabases in size, with a G + C content ranging from 32 to 46% mol (Javier-Lopez et al., 2022). Despite the discovery and characterization of a few keratinases (Kluskens et al., 2002; Kim et al., 2004; Godde et al., 2005; Lee et al., 2015), the feather-degrading potential of this group remains largely underexplored. In this context, the metabolic versatility of the *Fervidobacterium* group, their thermophilic features, and the increasing availability of thermostable proteases for the degradation of proteinaceous biowaste from agriculture and fisheries have huge potential for biotechnological and industrial applications.

Unraveling how biological systems function and elucidating key molecular mechanisms have become more attainable with the advent of genomics, transcriptomics, and proteomics (Armengaud, 2016). Next-generation proteomics can be applied to whole cells, as well as proteins secreted in the milieu (Armengaud et al., 2012), offering insights into enzymes and catalysts produced and exported by bacteria.

To the best of our knowledge, comparative proteomic and transcriptomic studies on *Fervidobacterium* representatives are lacking. To uncover novel enzymes involved in keratin degradation and gain insights into the associated metabolic pathways, the keratinolytic capabilities of all available *Fervidobacterium* strains were evaluated in this study, and the cellular and exo-proteomes of the most efficient strains grown with chicken feathers or glucose were analyzed. Finally, the transcriptomes of the three representative strains were established. This multiomics study provides a comprehensive overview of the functionality of this thermophilic bacterial genus and highlights its potential for keratin degradation.

## Materials and methods

### Experimental design and strains used in this work

All available isolates of the genus *Fervidobacterium* were included in this study: *Fervidobacterium pennivorans* T (CP050868) and *Fervidobacterium* sp. GSH (CP126982), both isolated and described by our research group in Bergen (Javier-Lopez et al., 2022); *Fervidobacterium pennivorans* DSM 9078^T^ (CP003260), *Fervidobacterium nodosum* Rt17-B1^T^ (CP000771), *Fervidobacterium* sp. DSM 13770 (CP126498), *Fervidobacterium islandicum* H-21^T^ (CP126499), *Fervidobacterium* sp. DSM 21710 (CP126500), *Fervidobacterium changbaicum* CBS-1^T^ (CP026721), *Fervidobacterium riparium* 1445t^T^ (CP009277) and *Fervidobacterium gondwanense* DSM13020^T^ (CP126501), acquired from the German Collection of Microorganisms and Cell Cultures (Leibniz Institute DSMZ)^1^; *Fervidobacterium thailandense* FC2004^T^ (CP140110), obtained through the Japan Collection of Microorganisms (JCM).^2^

The keratinolytic efficiencies of these strains were assessed based on their ability to degrade chicken feather keratin under anaerobic and thermophilic conditions. The experimental procedure included inoculating each strain into flasks containing plain mineral medium in the presence of a chicken feather and incubating the cultures at their optimal temperature based on the DSMZ guidelines. The cultures were monitored for 72 h, during which the integrity of the feathers was visually inspected. The keratinolytic strains underwent proteomic analysis to identify and quantify the potential enzymes involved in keratin degradation. Finally, transcriptome profiles of the three most efficient degradative strains were compared.

### Medium preparation and cultivation

The organisms used in this study were cultured using a uniform Mineral Medium for Freshwater bacteria (MMF). This medium consisted of an initial mineral formulation, trace elements, and vitamins supplemented with yeast extract, glucose, or a chicken feather as carbon sources. The mineral composition of MMF contained, per liter: NaCl, 1 g; MgSO_4_·7H_2_O, 0.3 g; KCl, 0.3 g; NH_4_Cl, 0.5 g; CaCl_2_·2H_2_O, 0.1 g; and KH_2_PO_4_, 0.3 g. Ten milliliters of the trace elements solution SL-10 (Koblitz et al., 2022) was added. The required amount of yeast extract was at least of 0.5 grams per liter (Friedrich and Antranikian, 1996). The mixture was sterilized by autoclaving at 121°C for 20 min. After cooling to 60°C, while flushing with sterile nitrogen gas, 10 mL of a vitamin solution was added. The composition of the vitamin solution was, per liter: 4-aminobenzoic acid, 8 mg; D(+) biotin, 2 mg; nicotinic acid, 20 mg; Ca-D(+) pantothenic acid, 10 mg; pyridoxamine·2HCl, 30 mg; thiamine dichloride, 20 mg; and vitamin B12, 10 mg. Furthermore, 2 mL of 25% cysteine-HCl solution was added as a reducing agent.

The pH was adjusted to 7.1 ± 0.1 with 1 M HCl, and the medium was transferred to sterile 20 mL serum flasks using the Hungate technique (Hungate, 1950; Miller and Wolin, 1974). Each flask was capped with butyl rubber corks and secured with aluminum seals. Glucose was added as a carbon source to a final concentration of 5 g/L from a sterile anaerobic stock using a syringe.

### Feather degradation assessment

To assess the keratinolytic efficiency of the organisms, the bacteria were incubated with MMF medium enriched with 0.5 g/L yeast extract and native chicken feathers (15 ± 5 mg). The feathers were washed with a solution of ethanol:methanol (1:1) to eliminate lipids, feces and other organic debris, and autoclaved (121°C, 20 min.), as previously described (Javier-Lopez et al., 2022).

The cultures were incubated at the optimal temperature for each strain, as recommended by the German Collection of Microorganisms and Cell Cultures (Leibniz Institute, DSMZ). These were: *F. changbaicum* CBS-1^T^ (80°C), *F. islandicum* H-21^T^ (65°C), *F. pennivorans* T (65°C), *F. pennivorans* DSM 9078^T^ (65°C), *F.* sp. GSH (65°C), *F.* sp. 21710 (70°C), *F. riparium* 1445^T^ (65°C), *F. thailandense* FC2004^T^ (80°C), *F.* sp. 13770 (65°C), *F. nodosum* Rt17-B1^T^ (70°C), *F. gondwanense* DSM 13020^T^ (65°C).

### Feather degradation assay

Although there is no standardized procedure for assessing keratin degradation (Qiu et al., 2020), one of the most reliable and straightforward methods is to calculate the difference in the weight of the substrate before and after incubation (De Oliveira Martinez et al., 2020). Accordingly, a quantitative feather degradation assay was designed to measure and compare the keratinolytic activity of the three most efficient strains, *F. pennivorans* T, *Fervidobacterium* sp. GSH and *F. islandicum* H-21^T^. The cultures were incubated for 72 h at the optimal temperature for each microorganism: 70°C for *F. pennivorans* T and *Fervidobacterium* sp. GSH and 65°C for *F. islandicum* H-21^T^. The experiment was performed in triplicate using chicken breast feathers as substrates. The feathers were weighed and placed aseptically in serum flasks that were previously gassed with sterile nitrogen. Next, 20 mL of sterile MMF medium enriched with yeast extract (0.5 g/L) was added to each flask and inoculated with 1 mL of a dense bacterial culture of the respective strains. After 0 (initial time), 12, 24, 36, 48, 60 and 72 h the cultures were aseptically filtered through a 5 mm pore Whatman syringe filter. The filter was washed with 70% ethanol to remove any residual media or biological material and dried at 65°C to a constant weight.

The weight of the remaining feathers was recorded, and the efficiency of feather degradation was calculated by comparing the final weight to the initial value.

### Genome annotations

Both proteomes and transcriptomes were mapped to the annotated genomes of the previously mentioned species. The Prokaryotic Genome Annotation Pipeline (PGAP) version 2023-05-17. build6771 (Tatusova et al., 2016)^3^ was used to predict genes and other features in the genomes of *Fervidobacterium* sp. GSH (CP126982) and *F. islandicum* H-21^T^ (CP126499). The genome annotation of *F. pennivorans* T (CP050868) already available in Genbank was used.

## Proteomics

### Sample preparation

The bacteria were inoculated into flasks containing 15 mL of sterile mineral MMF medium with 0.5% glucose or a chicken feather. Biological triplicates were then incubated for 24 (glucose culture) or 48 h (feather culture) at the optimal temperature for each strain. The cells were harvested by centrifuging at 4°C for 10 min at 5,000 g. The pellets were weighted and mixed with 6 μL of Laemmli buffer per milligram of pellet, while the supernatants were filtered through 0.2 μm disk filters to eliminate any remaining cells. The filtered fraction was then concentrated using Amicon centrifugal filters (10 kDa cut-off) for 25 min at 5,000 g and 4°C. Then, 100 μL of this concentrated supernatant was transferred to a tube along with 100 μL of Laemmli buffer. Both pellets and concentrated supernatants mixed with Laemmli buffer were denatured by boiling at 99°C for 10 min.

For each sample, a volume of 20 μL of extract was subjected to electrophoresis on a NuPAGE 4–12% Bis-Tris (Invitrogen) gel for 5 min at 200 V in MES buffer (Invitrogen). The proteins were then stained with ready-to-use Coomassie SimplyBlue SafeStain (Thermo Fisher Scientific), destained with MilliQ water washes, excised as a single polyacrylamide band, treated, and proteolyzed with trypsin, as previously described (Rubiano-Labrador et al., 2014).

### Tandem mass spectrometry and data interpretation

For each sample, the resulting tryptic peptides (15 out of 50 μL) were analyzed using tandem mass spectrometry with an Exploris 480 high-resolution tandem mass spectrometer (Thermo electron) coupled to a Vanquish Neo UHPLC in conditions similar to those previously described (Charlier et al., 2024). Briefly, peptides were desalted online with a PepMap 100 C18 pre-column and resolved on a reverse-phase Acclaim PepMap 100 C18 column (Thermo Fisher Scientific) at a flow rate of 250 nL/min with a 90 min gradient (5–25% B), followed by a 5 min gradient (25–40% B) with mobile phases A (0.1% HCOOH/100% H_2_O) and B (0.1% HCOOH/99.9%CH3CN). The mass spectrometer was operated in data-dependent acquisition mode with a Top20 strategy consisting of cycles of a full scan of peptide ions, followed by sequential selection of each of the 20 most intense precursors in the high-energy collisional dissociation cell, their fragmentation, and MS/MS scans of the resulting fragments. Only peptide ions with a charge state of 2+ or 3+ were selected for dissociation, with a dynamic exclusion of 10 s. Full-scan mass spectra from 350 to 1,500 *m/z* were acquired at a resolution of 120,000, whereas MS/MS scans were recorded at a resolution of 15,000. Peptide-to-spectrum assignment was performed with the Mascot software v2.5.1 (Matrix Science) against the annotated genome database of each specific strain.

Full-trypsin specificity with up to two missed cleavages allowed, fixed modification of carbamidomethylated cysteine, mass tolerances of 5 ppm for the precursors, and 0.02 Da for peptide fragments were selected as parameters. Methionine oxidation and asparagine and glutamine deamidation were selected as variable modifications. Peptide matches with a MASCOT peptide score below a *p*-value of 0.05 were considered. Proteins with at least two different peptides were selected, and their quantities were estimated using spectral counts. The false discovery rate for protein identification was <1%, as estimated using the MASCOT reverse decoy database option. Spectral counts were compared between conditions after standard normalization using the T-Fold method as previously described (Gouveia et al., 2020), selecting proteins that satisfied |T-fold| (≥1.5) and p-value (≤0.05) as significantly up-and downregulated. Volcano plots were drawn to visualize the results using the Ggplot2 (v 3.5.1) (Wickham, 2016) library in R.

### Functional analyses

The upregulated proteins identified in the studied strains were submitted to the Kyoto Encyclopedia of Genes and Genomes (KEGG) (Kanehisa and Goto, 2000; Kanehisa, 2019; Kanehisa et al., 2022), annotated and KO codes assigned with BlastKOALA and their global metabolism and pathways were identified, analyzed and compared using the Reconstruct tool in KEGG Mapper.

## Transcriptomics

### RNA isolation and purification

*Fervidobacterium pennivorans* T, *Fervidobacterium* sp. GSH and *F. islandicum* H-21^T^ were grown in triplicate with either glucose (0.5%) or native chicken breast feathers. After 18 (glucose and feather samples) or 40 h (feather cultures), the cells were harvested via centrifugation at 4°C for 10 min at 5,000 g, and total RNA was purified using the protocol described in the RNeasy Mini Kit from Qiagen. The RNA concentration was measured using a NanoDrop™ One/One^C^ spectrophotometer, and RNA integrity assessed using an Agilent 2,100 Bioanalyzer System (Agilent Technologies, California, USA) based on the calculation of the RNA integrity number (RIN). RNA samples were stored at −80°C until further analysis.

### Complementary (cDNA) synthesis, sequencing and assembly

The rRNA was depleted, and cDNA was synthesized and sequenced using Illumina technologies at Eurofins Genomics facilities, Constance, Baden, Germany.^4^ Low-quality reads (PHRED score < 30) and adapters were trimmed using CLC Workbench Genomics v23.0.5.^5^ The filtered reads were assembled and mapped to the annotated genomes of *F. pennivorans* T, *Fervidobacterium* sp. GSH and *F. islandicum* H-21^T^ with CLC Workbench Genomics v23.0.5, using the following parameters: mismatch cost = 2, insertion cost = 2, deletion cost = 3, length fraction = 0.8, and maximum number of hits per read = 10.

### Differential gene expression

Gene count normalization and differential gene expression analyses were performed using the DESeq2 package (v1.43.1) in Bioconductor hosted in R (version 2023.09.0 + 463). The analysis was performed separately for each strain, comparing total gene expression levels between the glucose and feather cultures at 18 and 40 h of incubation. After applying a variance-stabilizing transformation (VST), genes with a |fold change| > 1.5 and a false discovery rate (FDR) < 0.05 were considered significantly over-and under-expressed, respectively. The significant genes were subset and the results visualized using heatmaps drawn with the R package pheatmap v1.0.12.^6^

## Results

### Feather degradation assessment

The keratinolytic potential of all 11 *Fervidobacterium* strains available was assessed using feather degradation tests. Temperature conditions were set individually for each strain according to their optimal growth temperature. The results in Table 1 were obtained after incubating the cultures for 72 h in MMF medium using chicken feathers as the sole carbon source. Among the strains tested, only *F. nodosum* Rt17-B1^T^ and *F. gondwanense* DSM 13020^T^ showed no visible signs of feather degradation. *Fervidobacterium riparium* 1445t^T^, *Fervidobacterium* sp. 13770, and *Fervidobacterium thailandense* FC2004^T^ exhibited only partial feather degradation. The remaining six strains–*F. changbaicum* CBS-1^T^, *F. islandicum* H-21^T^, *F. pennivorans* T, *F. pennivorans* DSM 9078^T^, *F. pennivorans* GSH and *Fervidobacterium* sp. 21710–completely degraded feather within 72 h. *F. pennivorans* T, *F. pennivorans* GSH, and *F. islandicum* H-21^T^ were particularly efficient in degrading most of the feathers after only 48 h and were thus selected for further investigation in degradation assays and transcriptomics. Examples of complete, partial and negative feather degradation are displayed in Supplementary Figure S1.

**Table 1:** Overview of the feather degradation capacity of fervidobacteria.

Strain	Optimal growth temperature (°C)	Feather degradation
*Fervidobacterium changbaicum* CBS-1^T^	80	Positive
*Fervidobacterium islandicum* H-21^T^	65	Positive
*Fervidobacterium pennivorans* T	65	Positive
*Fervidobacterium pennivorans* DSM 9078^T^	65	Positive
*Fervidobacterium* sp. GSH	65	Positive
*Fervidobacterium* sp. 21710	70	Positive
*Fervidobacterium riparium* 1445t^T^	65	Partial
*Fervidobacterium thailandense* FC2004^T^	80	Partial
*Fervidobacterium* sp. 13770	65	Partial
*Fervidobacterium nodosum* Rt17-B1^T^	70	Negative
*Fervidobacterium gondwanense* DSM 13020^T^	65–68	Negative

**Figure 1:**
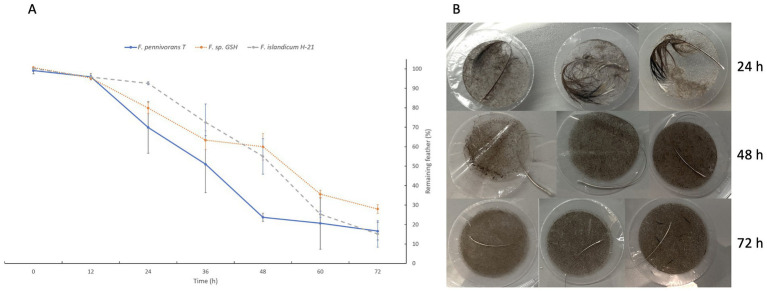
Quantitative feather degradation assay showing the difference in weight measured in percentage of remaining feathers after 12, 24, 36, 48, and 72 h of incubation. **(A)** Assay results for *Fervidobacterium pennivorans* T (blue), *Fervidobacterium pennivorans* GSH (orange), and *Fervidobacterium islandicum* H-21^T^ (gray). **(B)** Feather degradation by *F. pennivorans* T after 24, 48, and 72 h of incubation, as revealed by filtering through a 5 mm-pore syringe filter (triplicates).

A quantitative degradation assay was performed to quantify and compare the keratinolytic activity of the three most efficient strains of fervidobacteria, namely *F. pennivorans* T, *F. pennivorans* GSH, and *F. islandicum* H-21^T^. Figure 1 shows the percentage of feather material remaining after 12, 24, 36, 48, 60 and 72 h of incubation. Feather weight loss was expressed as a percentage of the original weight. By the 12 h mark, there was almost no variation in weight loss, and the degradation became clearly noticeable after this time point. After 24 h, *F. pennivorans* T had broken down more than 30 ± 13% of the feather material, and *F. pennivorans* GSH had degraded approximately 20 ± 3%. The slowest strain at this time point was *F. islandicum* H-21^T^, with around 7 ± 1% degradation. At this time point, the degradation rate of all three strains significantly increased until 60 h, then slowed, except for *F. pennivorans* T, the most efficient strain, which reached a plateau after only 48 h. At 60 h, a stationary phase in feather degradation was reached for all three strains, but feather degradation could still be measured until the end of the experiment, at 72 h. At the conclusion of the assay, *F. islandicum* H-21^T^ and *F. pennivorans* T had degraded most of the feather, with only 15 ± 7% and 17 ± 5% remaining, respectively. *F. pennivorans* GSH could only break down until 28 ± 2% of the feather after a 72-h incubation.

## Shotgun proteomics

### Differential analysis

The six most active keratinolytic fervidobacteria (*F. changbaicum* CBS-1^T^, *F. islandicum* H-21^T^, *F. pennivorans* T, *F. pennivorans* DSM 9078^T^, *F. pennivorans* GSH and *Fervidobacterium* sp. 21710) and *F. gondwanense* DSM 13020^T^ as a non-degradative (negative control) strain were subjected to a dual shotgun proteomic study, in which the cellular proteome and exoproteome were established (a list of proteins with statistical analysis is available as a separate Excel file in File S2). More than 80% of the approximately 2,000 proteins detected in each of the seven proteomes were identified, most of which were identified in the cellular proteome, with more than 1,250 proteins identified per strain (Table 2), whereas only approximately 200 proteins on average belonged to the exoproteome fraction. Approximately 160 proteins were found upregulated in the feather cultures of all strains, most of which were in the cellular fraction. The number of downregulated proteins identified was notably higher, with more than 200 proteins in each strain, except for *Fervidobacterium* sp. 21710, which had only 151 downregulated proteins. Surprisingly, the non-keratinolytic bacterium *F. gondwanense* DSM13020^T^ showed the highest number of upregulated proteins, with a total of 76 proteins identified.

**Table 2:** Proteomics overview of the number of proteins detected with at least 2 peptides and numbers of significantly up-and downregulated proteins.

		Total identified		Upregulated secreted	Downregulated secreted	Upregulated cellular	Downregulated cellular	Reductases					Peptidases				
Strain	Total	Exoproteome	Cellular proteome					Total	Spectral Count		Identified	Upregulated	Total	Spectral Count		Identified	Upregulated
									Glc	F				Glc	F		
*F. pennivorans* T	1,828	177	1,310	22	15	148	509	84	2,416	1,311	70	11	49	1,350	701	39	8
*Fervidobacterium* sp. GSH	1,871	173	1,389	7	7	217	401	85	3,550	1,869	76	15	52	1,436	939	40	10
*F. islandicum* H-21^T^	1,987	90	1,430	2	5	123	289	89	1,757	1,157	76	21	51	1,143	734	45	3
*F. pennivorans* DSM9078^T^	1,947	279	1,436	10	25	110	418	102	3,188	1,784	90	10	56	1,503	1,038	44	11
*Fervidobacterium* sp. 21710	1,973	362	1,462	5	90	143	151	97	2,216	2,066	90	14	53	919	1,057	40	9
*F. changbaicum* CBS-1^T^	1,980	123	1,256	11	20	116	393	91	1,972	1,292	70	20	43	1,225	760	32	7
*F. gondwanense* DSM13020^T*^	1,984	206	1,397	76	19	146	447	92	2,978	1,116	76	26	50	1,347	511	44	16

The total number of enzymes annotated in the genomes, the detected proteins in the analysis, and the number of significantly upregulated reductases and peptidases are shown. The total spectral count in the glucose (Glc) and feather (F) cultures is also included. Proteins with a spectral count >50 after normalization were considered. * While *F. gondwanense* is unable to grow on feather keratin, its genes were regulated in its presence/absence.

**Figure 2:**
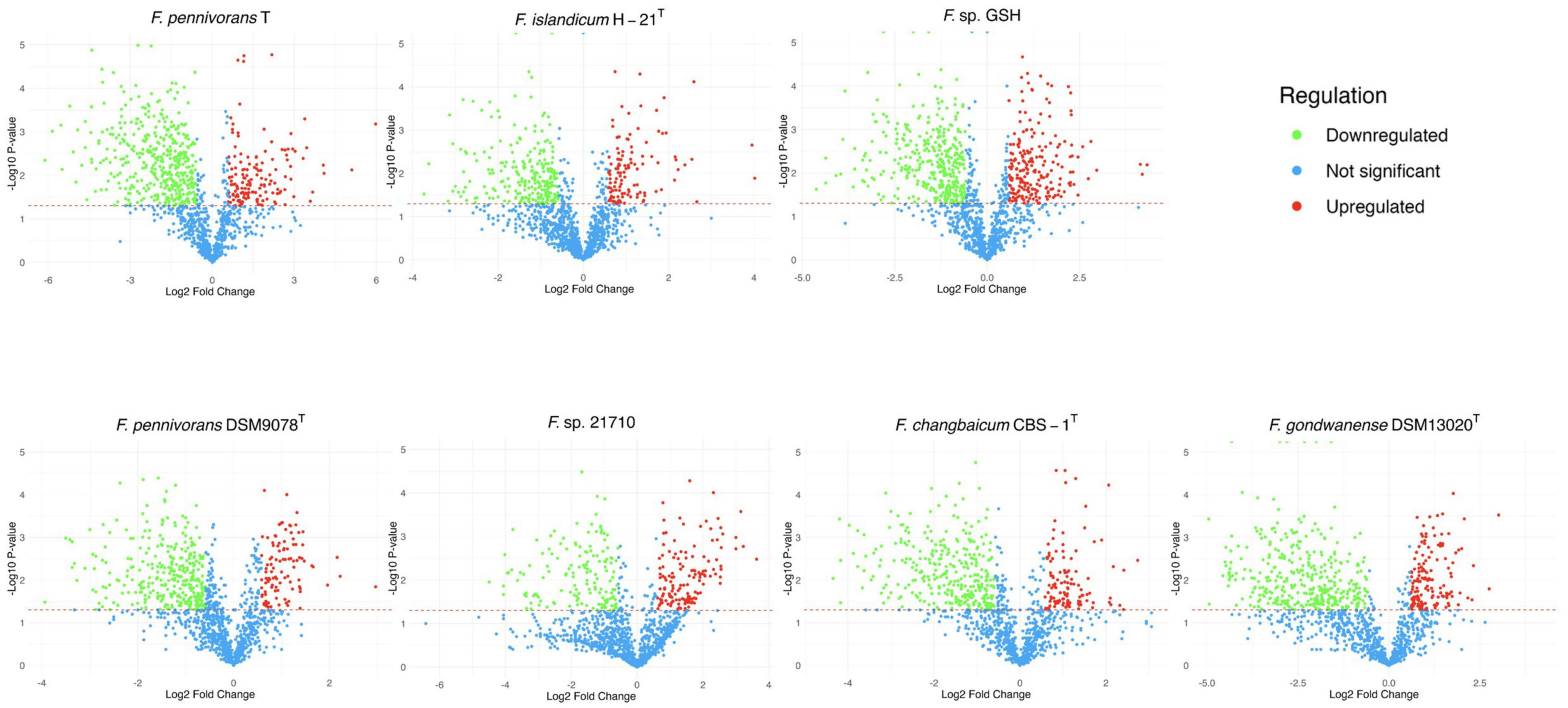
Volcano plot representations of the proteins identified in the cellular proteome of the described strains. The green dots represent the under-detected proteins in the feather condition compared to the glucose condition, and the red dots indicate the over-detected ones, with a |fold| threshold of 1.5. The statistical threshold was set at 0.05, with those proteins not significantly modified in terms of abundance, i.e., with higher *p*-values, as well as non-regulated proteins, indicated in blue.

Differential analysis is shown in Figure 2 in the form of volcano plots, where protein abundances were compared between feather and glucose conditions. Proteins were classified as upregulated or downregulated based on a |fold change| threshold of 1.5 and a significance level (*p*-value) below 0.05. Across all strains, the statistically underrepresented proteome was more abundant, indicating that the proteome of cells grown in feather conditions was less diverse and, thus, more specialized than the proteome of cells grown on glucose. For most strains, the number of significantly downregulated proteins was between two and three times more abundant in the feather condition than in the glucose condition, except for *Fervidobacterium* sp. 21710, which had almost the same number of differential proteins in both fractions.

Genome annotation of these strains revealed that each strain encoded approximately 100 reductases and 50 peptidases. Proteomic analysis identified >70 reductases and at least 40 peptidases, except for *F. changbaicum* CBS-1^T^ and *F. pennivorans* T, with 32 and 39 detected peptidases, respectively (Table 2). The strain with the highest number of upregulated reductases and peptidases was again the non-keratinolytic strain *F. gondwanense* DSM13020^T^, with 26 and 16, respectively, approximately 35% of the total of both types of enzymes. Among the keratinolytic strains, *F. islandicum* H-21^T^ showed the highest number of upregulated reductases (21), accounting for 28% of the total identified reductases. This contrasts with the sparse number of differentially detected peptidases, with only three being the lowest among all the strains studied. The fraction of upregulated reductases in the other strains ranged from 10 (11% of the total identified) for *F. pennivorans* DSM9078^T^ to 20 (29% of the total identified) for *F. changbaicum* CBS-1^T^. The number of upregulated peptidases was lower, even more so than that of the reductases, ranging from 11 in *F. pennivorans* DSM9078^T^ (25% of the total identified) to 7 in *changbaicum* CBS-1^T^ (22% of the total identified). The accession numbers of the upregulated peptidases and reductases identified in each strain are listed in Supplementary Table S1.

### KEGG functional analysis

The sequences of significantly upregulated proteins in *F. pennivorans* T, *Fervidobacterium* sp. GSH and *F. islandicum* H-21^T^ were analyzed using the BlastKOALA (KEGG Orthology and Links Annotation) tool. A total of 111 proteins (69.8% of sequences) from the dataset of *F. pennivorans* T, 137 (62.8% of sequences) from *Fervidobacterium* sp. GSH, and 81 (64.8%) from *F. islandicum* H-21^T^ were successfully annotated. The largest KEGG category was related to the carbohydrate metabolism pathways, with 19 entries from *F. pennivorans* T and 26 from *Fervidobacterium* sp. GSH, and 18 from *F. islandicum* H-21^T^. Additionally, amino acid metabolism pathways included 15 proteins from *F. pennivorans* T and 11 proteins from *Fervidobacterium* sp. GSH and eight from *F. islandicum* H-21^T^ (Supplementary Figure S2).

### Keratinolytic and non-keratinolytic strains comparison

The sequences of the upregulated peptidases identified in the most active strains, *F. pennivorans* T, *Fervidobacterium* sp. GSH and *F. islandicum* H-21^T^, were blasted against the proteome of *F. gondwanense* DSM13020^T^, a non-keratinolytic member of *Fervidobacterium,* using the protein sequences of *F. pennivorans* T. A total of 20 proteins were considered in this analysis. Six of them (QIV79356.1, QIV78721.1, QIV79147.1, QIV78659.1, QIV78935.1 and QIV78782.1) were not detected in *F. gondwanense*. The amino acid identity of these proteins ranged from 22.7 to 88.6%, with a median value of 80.4%. Interestingly, orthologues of three peptidases previously categorized as “true” keratinases (Qiu et al., 2020) (QIV78374.1, QIV78926.1 and QIV78937.1) were strongly downregulated in *F. gondwanense*. Furthermore, the amino acid identity of QIV78926.1 and QIV78937.1 compared to the orthologues of *F. gondwanense* was 55.3 and 54.9%, respectively (Table 3).

**Table 3:** Peptidase comparison across the most active strains and the non-keratinolytic F. gondwanense*.

Functional annotation	*Fervidobacterium pennivorans* T	*Fervidobacterium* sp. GSH	*Fervidobacterium islandicum* H-21ᵀ	*Fervidobacterium gondwanense* DSM13020ᵀ	% Identity *F. pennivorans* T *F. gondwanense*	Fold Change *F. gondwanense* DSM13020ᵀ
M48 family metallopeptidase	**QIV78659.1**	XEY11950.1	XEY09979.1	XEY03790.1	63.60%	Undetected
Zinc metallopeptidase	QIV78721.1	**XEY11881.1**	XEY09904.1	XEY03712.1	86.10%	Undetected
S9 family peptidase	QIV78782.1	XEY11827.1	XEY09860.1	XEY05362.1	22.70%	Undetected
M42 family metallopeptidase	**QIV78935.1**	XEY11656.1	**XEY09629.1**	XEY04549.1	40.20%	Undetected
Peptidase M55	QIV79147.1	XEY12292.1	XEY10359.1	XEY04413.1	74.80%	Undetected
M42 family metallopeptidase	QIV79356.1	XEY13256.1	XEY11379.1	XEY04549.1	88.00%	Undetected
Aminopeptidase	QIV78194.1	XEY12978.1	**XEY10989.1**	**XEY04186.1**	82.30%	27.70
Carboxypeptidase M32	**QIV78128.1**	XEY13038.1	XEY11065.1	**XEY04784.1**	66.70%	7.33
Dipeptidase PepV	QIV78327.1	XEY11555.1	XEY09511.1	**XEY05364.1**	83.20%	7.29
M42 family metallopeptidase	QIV78118.1	XEY13048.1	XEY11075.1	**XEY04774.1**	73.70%	4.00
Do family serine endopeptidase	**QIV79267.1**	XEY11596.1	XEY09564.1	**XEY03519.1**	83.40%	2.52
ATP-dependent protease subunit HslV	**QIV78519.1**	XEY13122.1	XEY11160.1	**XEY05042.1**	82.60%	1.54
M42 family metallopeptidase	**QIV79343.1**	XEY11657.1	**XEY09630.1**	XEY04818.1	84.20%	−2.43
Beta-aspartyl-peptidase	QIV78699.1	XEY11904.1	**XEY09928.1**	XEY03738.1	71.40%	−2.59
Type I methionyl aminopeptidase	**QIV78895.1**	XEY11702.1	XEY09672.1	XEY04868.1	78.50%	−3.00
***S8 family serine peptidase***	*QIV78926.1*	*XEY11666.1*	***XEY09639.1***	*XEY04828.1*	55.30%	−3.67
***S8 family serine peptidase***	***QIV78937.1***	*XEY11654.1*	***XEY09623.1***	*XEY03572.1*	54.90%	−6.82
S41 family peptidase	QIV79051.1	**XEY12183.1**	**XEY10221.1**	XEY04539.1	84.20%	−9.33
ATP-dependent Clp protease ATP-binding subunit ClpX	**QIV79179.1**	XEY11435.1	XEY11261.1	XEY04710.1	83.90%	−9.67
***S8 family peptidase***	***QIV78374.1***	*XEY11509.1*	***XEY09446.1***	*XEY05306.1*	88.60%	−24.80

*All upregulated peptidases (bold characters) detected either in *F. pennivorans* T, *Fervidobacterium* sp. GSH and *F. islandicum* were included. Orthologues of known keratinases are italicized.

## Transcriptomics

**Figure 3:**
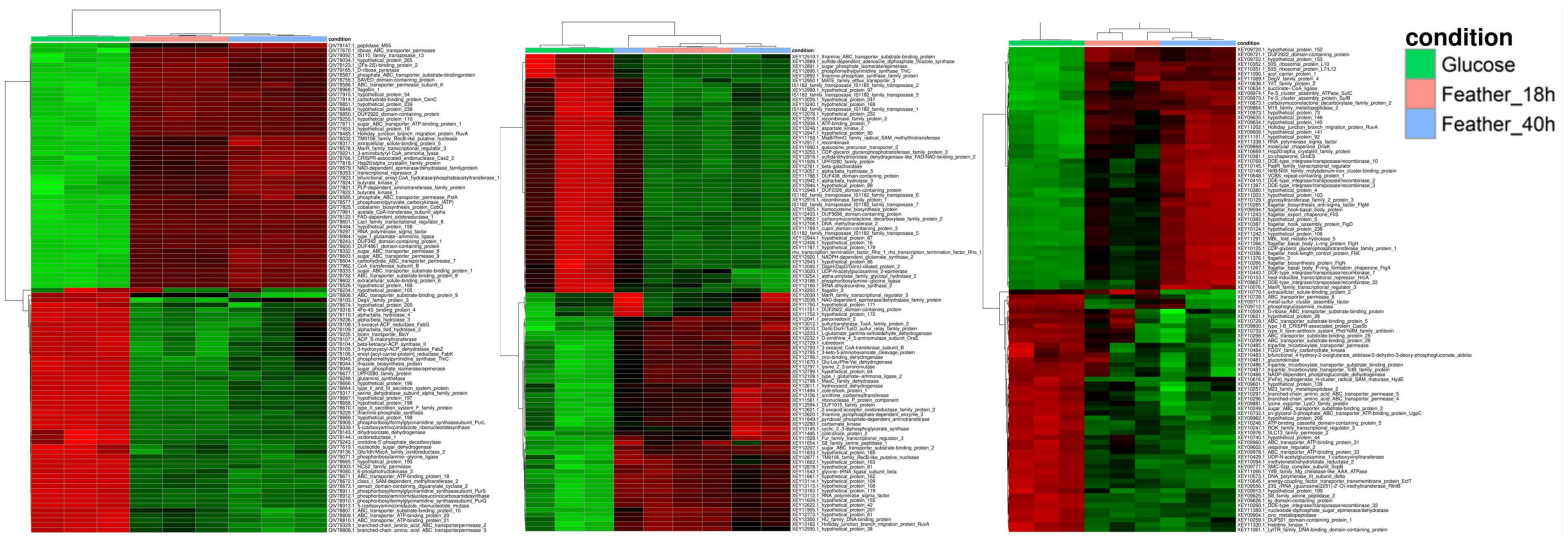
Heatmap representation with the most promiment genes among the significantly expressed features of *F. pennivorans* T, *Fervidobacterium* sp. GSH, and *F. islandicum* H-21^T^. The columns represent the different samples, corresponding to the substrates used in the experiment: glucose and chicken feathers. The rows indicate the relative abundance of the genes, from high (red) to low expression (green).

Deep mRNA sequencing (62.3 GB data) led to high coverage per sample: 2,813 × for *F. pennivorans* T and 2,361 × for *Fervidobacterium* sp. GSH, and 2,141 × for *F. islandicum* H-21^T^. More than 90% of the reads mapped to annotated genomes for all samples, except for two replicates with slightly lower values. In general, the broken pairs remained below 2% for all the samples (Supplementary Tables S2–S4). Following normalization and statistical analysis with DESeq2, 1,716 genes (FDR < 0.05) were identified in *F. pennivorans* T and 1,398 in *Fervidobacterium* sp. GSH, and 953 in *F. islandicum* H-21^T^. Less than half of these genes were overexpressed (Fold >1.5) in *F. pennivorans* T and *Fervidobacterium* sp. GSH (728 and 665, respectively), whereas 502 genes were overexpressed in *F. islandicum* H-21^T^ compared with the feather- and glucose-grown cells. Heatmaps drawn using the 50 most overexpressed and the 50 most underexpressed genes of the three strains are shown in Figure 3, which shows strong genetic reprogramming in the three strains to adapt to the carbon source. Furthermore, the expression of some genes was higher after 18 h of growth, whereas others were more abundant after 40 h, indicating that some genes were switched on and off earlier than others when these strains were growing with chicken feathers.

### Peptidases and oxidoreductases

The expression of 47 genes annotated as peptidases or proteases was significantly (FDR < 0.05) modified in the *F. pennivorans* T transcriptome, 19 of which were overexpressed in either the 18 or 40 h samples.

Several enzymes with keratinolytic potential have been identified, including metallopeptidases (QIV78118.1, QIV79325.1, QIV79356.1, QIV79147.1, QIV78781., QIV78721.1, QIV78572.1, and QIV79192.1), carboxypeptidases (QIV79319.1), and serine peptidases (QIV78782.1, QIV78937.1). For *Fervidobacterium* sp. GSH, 36 peptidases or proteases were identified in the transcriptome. A total of 21 of these enzymes were significantly overexpressed in the 18 or 40 h samples. Among these enzymes, metallopeptidases (XEY13048.1, XEY13256.1, XEY12292.1, XEY11656.1, XEY12216.1, XEY12589.1 and XEY12665.1) or serine peptidases (XEY11827.1, XEY11654.1 and XEY11509.1). Finally, 27 of these enzymes were identified in the proteome of *F. islandicum* H-21^T^ (FDR < 0.05), 14 of which were overexpressed in the samples harvested after 18 or 40 h. Several overexpressed metallopeptidases (XEY11075.1, XEY09864.1, XEY10359.1 and XEY10721.1) and serine peptidases (XEY09860.1 and XEY09564.1) were also found in this bacterium. Notably, three of these genes were overexpressed in feather-grown cells of all three strains: two metallopeptidases of the M42 and M55 subfamilies, a serine S9 family protease, and the dipeptidase PepV. In addition, a gene annotated as an ATP-dependent Clp protease ATP-binding subunit was overexpressed in all three strains.

Supplementary Table S5 shows the most overexpressed and most upregulated peptidases, proteases and oxidoreductases detected across the analyzed strains in both Transcriptomics and Proteomics analyses. This table also includes the fold-changes, Enzyme Commission (EC) numbers and the amino acid percentage identity.

Only a fraction of the statistically significant peptidases and proteases detected in these three strains is overexpressed in the presence of feathers, suggesting that not all peptidases of these strains are necessary for keratin degradation. Some of them had high expression levels only at one of the kinetic points, that is, after 18 or 40 h of incubation, with only a few of these genes found to be active throughout the entire incubation period in feather cultures.

Keratin degradation begins with the action of different oxidoreductases. A total of 76 of these genes were detected in the transcriptome of *F. pennivorans* T, 38 of which were overexpressed. Regarding *Fervidobacterium* sp. GSH, 39 overexpressed reductases within a total of 65 significant ones were identified, a similar number compared to *F. pennivorans* T. Finally, 46 reductases were identified among the significant genes in *F. islandicum* H-21^T^, 29 of which were overexpressed in the feather cultures, a lower count compared with the other two strains. Seven reductases were overexpressed in all three strains, suggesting that they were important for the reaction. These enzymes were identified as Glu/Leu/Phe/Val dehydrogenase, 2-oxoacidacceptor oxidoreductase subunit alpha, NAD (P)/FAD-dependent oxidoreductase, NADH-quinone oxidoreductase subunit NuoF, SDR family oxidoreductase, L-threonine 3-dehydrogenase, 2,4-diaminopentanoate dehydrogenase, and 2-hydroxyacid dehydrogenase.

Notably, more than half of the reductases of *F. pennivorans* T and *Fervidobacterium* sp. GSH were switched on after only 18 h of incubation, whereas only a few were overexpressed after 40 h of incubation; thus, they can be considered late-induced reductases. This suggests that more reductases are activated during the early stages of bacterial growth. *F. islandicum* H-21^T^ did not exhibit a clear pattern. Most of the overexpressed reductases identified in this strain were found after 48 h of incubation, with only a few that could be considered early induced reductases. This behavior may also explain the longer time required by this bacterium to adapt to changes in the carbon source and fully degrade feathers.

Among the most abundant proteins in the supernatants of feather cultures, both peptidases and reductases were detected, along with other enzymes such as specific ABC substrate-binding transporters. Additionally, the proteins found upregulated in the three most active strains and their functional annotations are available in a separate Excel file as Supplementary Table S6.

## Proteomics and transcriptomics integration

**Figure 4:**
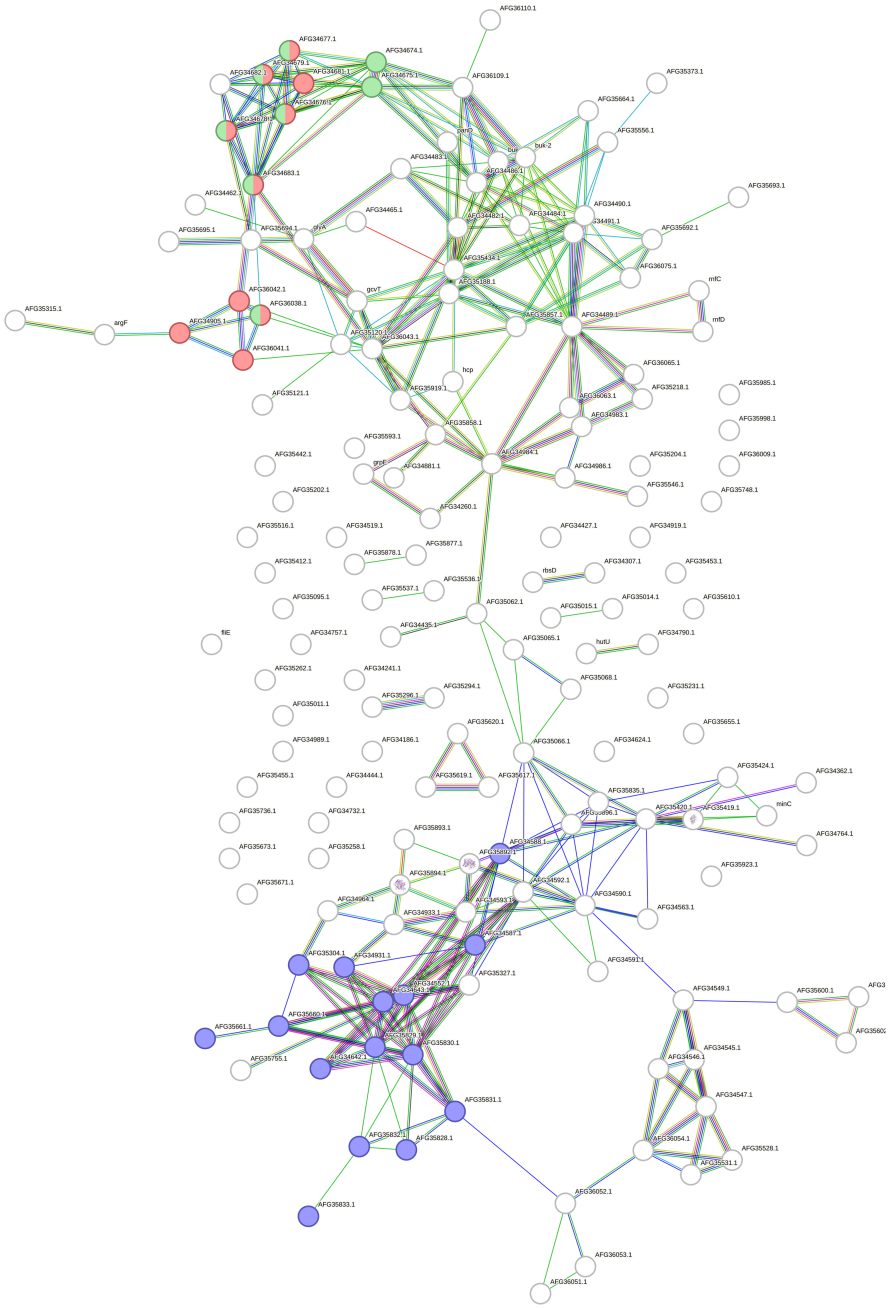
Protein and gene network made with STRING. The network contains all the upregulated proteins and genes identified in *F. pennivorans* T when growing the bacterium with a chicken feather, compared to cultures grown with glucose. The circles represent the features, and the lines indicate the connections found by STRING. Features related to lysine degradation are colored in red (STRING cluster CL:3375) and green (KEGG pathway fpe00310), and those assigned to extracellular solute binding (STRING cluster CL:2832) are colored in blue.

A total of 164 overrepresented features of *F. pennivorans* T were identified by combining the results of transcriptomic and proteomic analyses. The interactions between these features were analyzed using the STRING tool. A network with 325 edges with an estimated *p*-value lower than 1.0e^−16^ was obtained. This network indicates a high number of statistically relevant interactions, that is, biologically relevant connections. Among the clusters identified using STRING, a group of features related to lysine degradation stood out, with 10 and 8 features annotated in the STRING cluster CL: 3375 (FDR = 0.03) and the KEGG pathway fpe00310 (FDR = 0.003), respectively. In addition, 15 features were assigned to the STRING cluster CL: 2832 (FDR = 0.03), which is related to bacterial extracellular solute binding (Figure 4).

**Figure 5:**
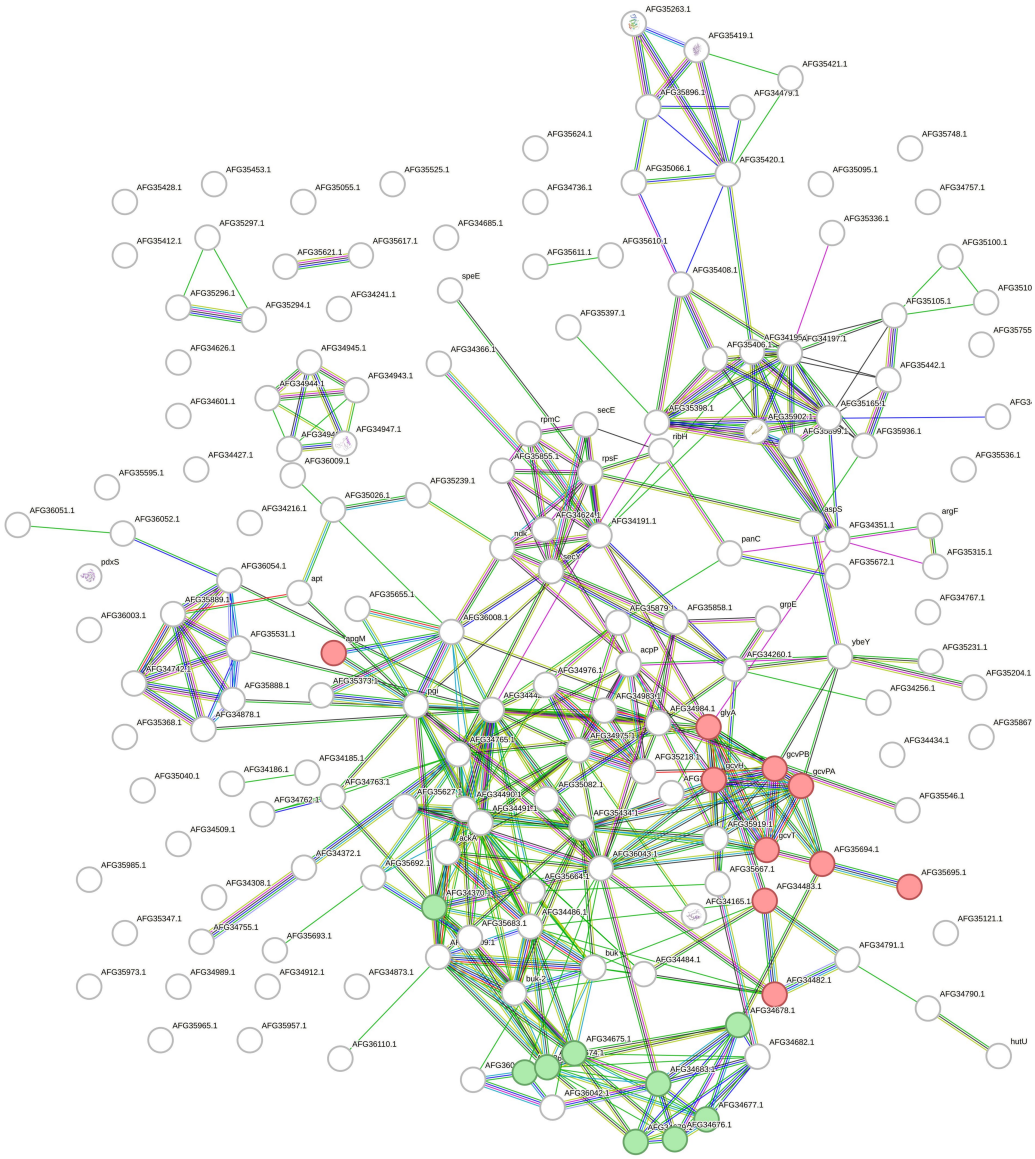
STRING network built with the upregulated and overexpressed features identified in *F*. sp. GSH. Overexpressed genes and upregulated proteins when growing the cells with chicken feathers were used. Green colored circles correspond to features in the lysine degradation pathway (fpe00310) and the red ones to glycine, serine and threonine metabolism (fpe00260), both from KEGG database.

For *Fervidobacterium* sp. GSH, lysine degradation (fpe00310, FDR 0.00047), and glycine, serine, and threonine metabolism (fpe00260, FDR 0.0017) pathways were significant. Additionally, several biological processes annotated in the Gene Ontology database were detected, some of which were related to keratin and protein degradation processes. Overall, 384 edges were created in the network over the expected number of 209 edges with a p-value lower than 1.0e^−16^ (Figure 5).

**Figure 6:**
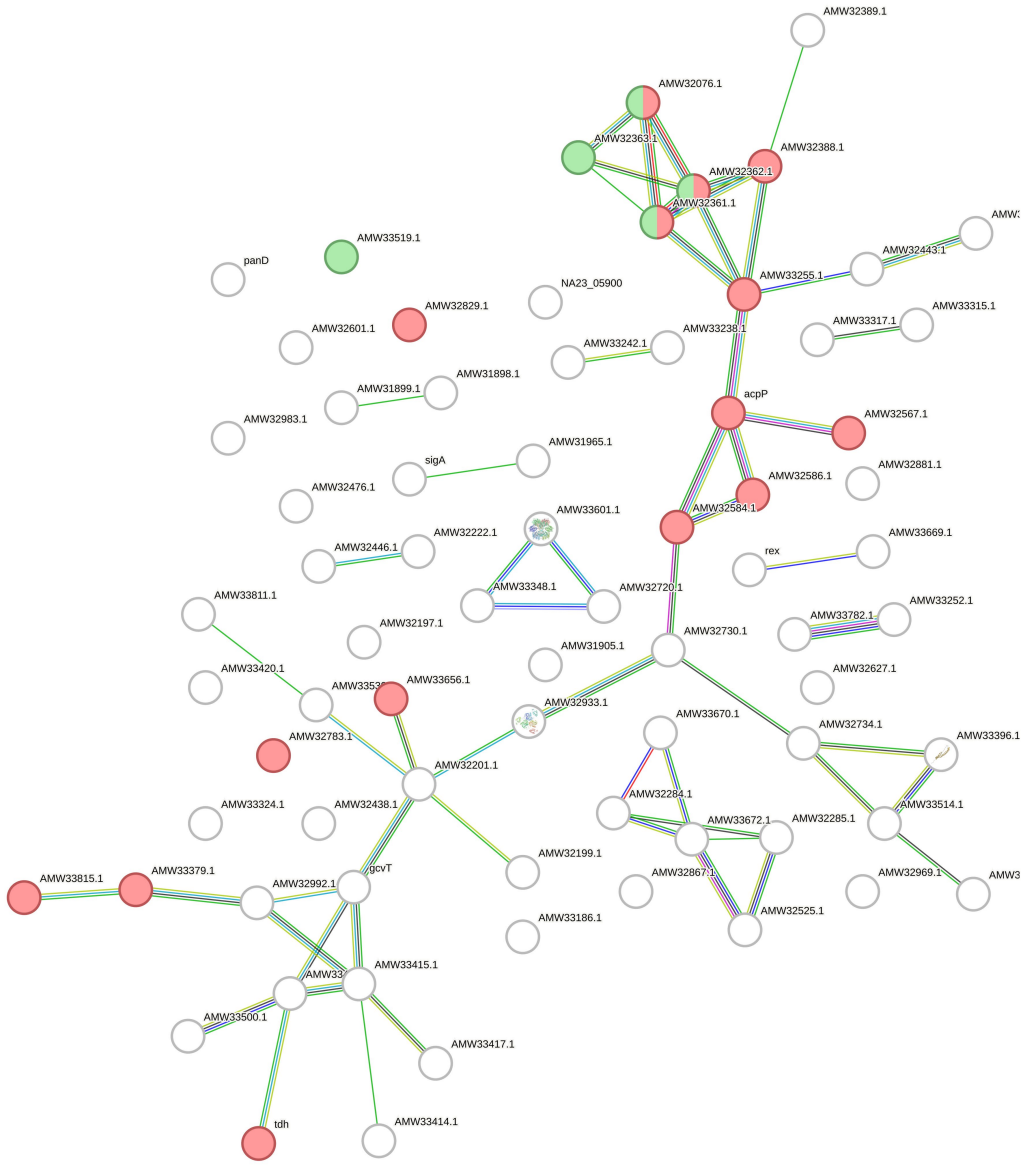
STRING network with the upregulated proteins and overexpressed genes of *F. islandicum* H-21^T^. The circles represent the features of the bacterium annotated by STRING and the lines indicate the interactions among them. The features of the CL:934 cluster (Mixed, incl. Oxidoreductase activity, and Butanoate metabolism, red) and of the fia00310 KEGG Pathway (Lysine degradation, green) are highlighted.

Finally, for *F. islandicum* H-21^T^, 74 selected features provided a STRING network with 60 edges (*p* = 7.71e-05). In this case, 11 features of the oxidoreductase activity STRING-related cluster (CL: 934) were annotated with a significant FDR of 0.0207. Five features of the lysine degradation pathway in KEGG (fia00310) were identified and annotated with an FDR of 0.017 (Figure 6).

### A model of keratin degradation

**Figure 7:**
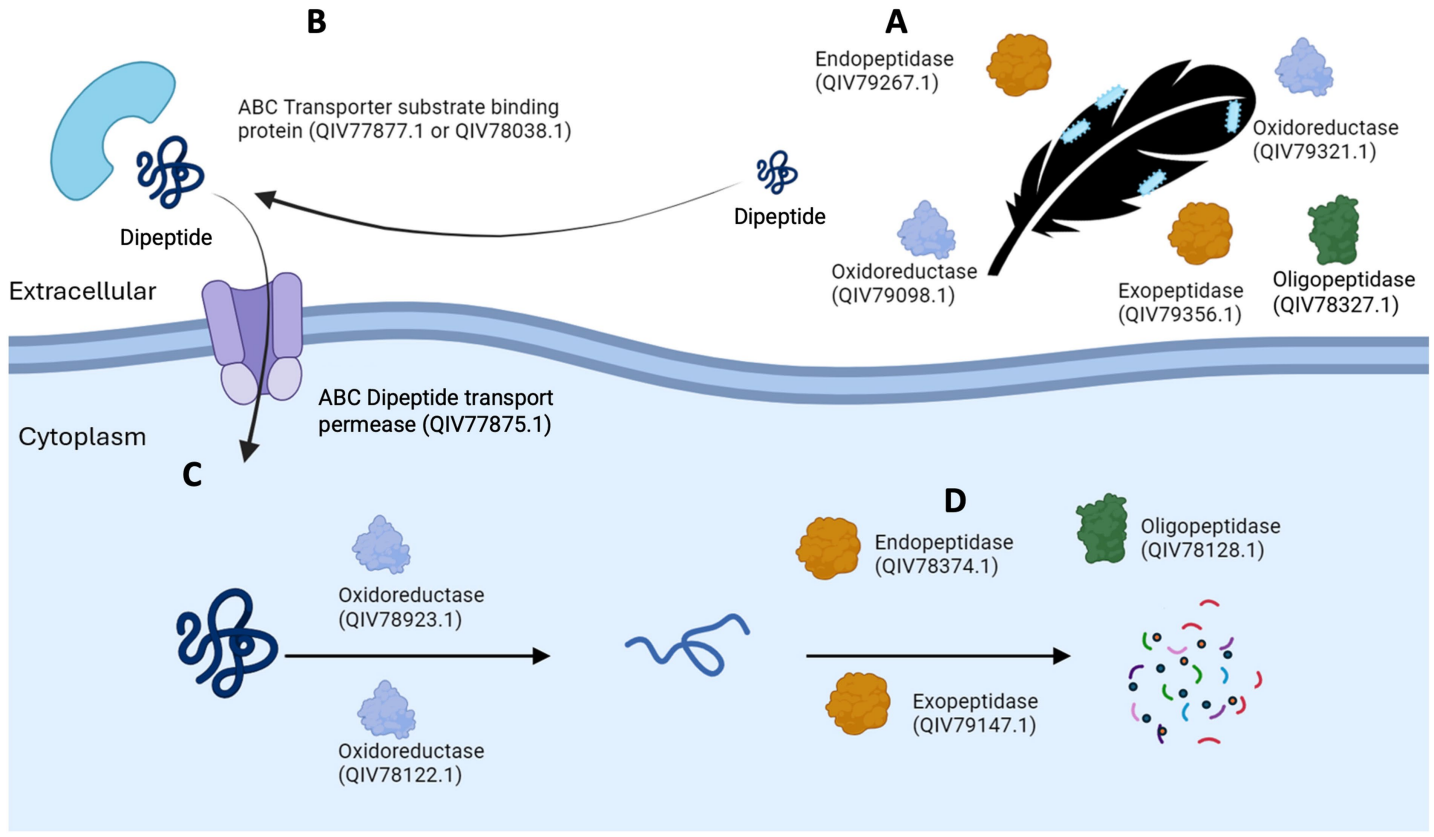
A model of keratin degradation by *Fervidobacterium,* made with upregulated enzymes identified in *F. pennivorans* T. Candidate peptidases and oxidoreductases start the reaction outside the cell **(A)**, specific peptide binding proteins **(B)** and transporters introduce smaller peptides into the cytoplasm **(C)**, where the combined action of cellular oxidoreductases, exo-, endo-and oligopeptidases complete the reaction.

A hypothetical model for keratin degradation is presented in Figure 7, showing overrepresented candidate oxidoreductases, peptidases and specific peptide binding proteins and transporters of *F. pennivorans* T in the feather cultures. The location (extra-or intracellular) of the enzymes reflects their presence in the upregulated exo-or cellular proteome. The exo-and endopeptidase assignation is based on MEROPS database. The process starts when cells of *F. pennivorans* T bind to the feather. At this point, cell-bound and extracellular features would start the reaction. Both extracellular oxidoreductases (QIV79098.1, QIV79321.1), exo- (QIV79356.1), endo- (QIV79267.1) and oligopeptidases (QIV78327.1) that could potentially participate in this step are shown. Smaller peptides would be captured by specific substrate binding proteins (QIV77877.1, QIV78038.1) and transferred into the cytoplasm by specific permeases [QIV77875.1, a dipeptide transporter, as predicted by sequence comparison (Consortium, 2024)]. Then, cellular oxidoreductases (QIV78122.1, QIV78923.1) would reduce the disulfide bonds and a combined action of cellular exo- (QIV79147.1), endo- (QIV78374.1) and oligopeptidases (QIV78128.1) would decompose the peptides and finish the degradation.

## Discussion

Keratin is a robust and recalcitrant protein that is slowly degraded despite its high occurrence in nature. While several microorganisms can break down feather keratin, only a few are thermophilic, and a minority are anaerobic (Daroit and Brandelli, 2014; Sahni et al., 2015; Srivastava et al., 2020). This study assessed the keratinolytic capabilities of all available isolates in the genus *Fervidobacterium*, a group of bacteria known to count with keratinolytic members, and investigated and compared their transcriptomes and proteomes when grown with glucose or chicken feathers to highlight the key molecular players for feather degradation.

Among the studied organisms, only *F. nodosum* Rt17-B1^T^ and *F. gondwanense* DSM13020^T^ showed no evidence of keratin degradation. *F. riparium* 1445t^T^, *F. thailandense* FC2004^T^ and *Fervidobacterium* sp. 13770 exhibited partial activity, as they broke down a portion of the feathers with which they were cultured, but the degradation halted at some point, rendering the reaction incomplete. The six remaining strains, *F. changbaicum* CBS-1^T^, *F. islandicum* H-21^T^, *F. pennivorans T*, *F. pennivorans* DSM9078^T^, *Fervidobacterium* sp. GSH and *Fervidobacterium* sp. 21710 could degrade feather keratin completely within 72 h. Thus, 6 out of 11 *Fervidobacterium* strains displayed clear keratinolytic activity, demonstrating the potential of this group of bacteria for applications in feather and keratin degradation reactions. Among the aforementioned strains, three showed high activity: *F. islandicum* H-21^T^, *F. pennivorans* T, and *Fervidobacterium* sp. GSH, being *F. pennivorans* T particularly efficient as it could degrade a chicken feather almost completely after 48 h at 70°C.

Between 70 and 92% of the total proteins in bacteria were identified using label-free shotgun proteomics. Of these, 10–15% were significantly more abundant in feather cultures, most of them detected in the cellular proteome, indicating that this fraction of proteins was upregulated in the presence of feathers. Surprisingly, *F. gondwanense* DSM13020^T^ had a high number of upregulated proteins despite its inability to damage the integrity of feathers. This indicated that the growth of this bacterium halted when the yeast extract supplement in the culture was depleted. A comparison of the upregulated peptidases across the most active strains with the peptidases of *F. gondwanense* DSM13020^T^ showed that several of these enzymes in the latter were downregulated in the feather cultures, suggesting deficiencies in the regulation of these enzymes in *F. gondwanense* DSM13020^T^, which may explain the inability of this organism to attack feather keratin. In particular, the “true” keratinases QIV78374.1, QIV78926.1 and QIV78937.1 were strongly downregulated in *F. gondwanense* whereas clearly upregulated in the keratinolytic strains. These three enzymes shared a sequence identity of 88.6, 54.9 and 55.3%, respectively and compared with their orthologues in *F. pennivorans* T, suggesting potential deficiencies in their sequence and/or structure, particularly in QIV78926.1 and QIV78937.1. So, these three enzymes may be required for successful feather degradation and can be good candidates to be further explored as keratinolytic enzymes. Furthermore, genus *Fervidobacterium* has undergone several horizontal gene transfer events (Nelson et al., 1999; Frock et al., 2010; Cuecas et al., 2017) and shows an intricated evolutionary history (Javier-López et al., 2024), which might have resulted in the loss or truncation of some of these enzymes.

The pathways involved in keratin degradation remain to be elucidated. It has been hypothesized that keratin degradation cannot be completed by a single hydrolytic enzyme, so keratinolytic organisms may possess and activate multiple and different enzymes to effectively decompose this recalcitrant molecule (Huang et al., 2015; Fellahi et al., 2016). Thus, current hypothesis is that this reaction requires at least oxidoreductases to cleave the disulfide bonds of the molecule and endo-and exopeptidases to hydrolyze the exposed peptide bonds (Lange et al., 2016; Shavandi et al., 2017; Qiu et al., 2020). Furthermore, it has been previously shown that feather degradation may start with physical binding of the bacteria to the surface of the feather, something already described in *Fervidobacterium* (Kang et al., 2020; Javier-Lopez et al., 2022) and other taxa (Jeong et al., 2010), highlighting the importance of intracellular and membrane-bound enzymes in the process.

Although fervidobacteria possess a similar total number of these enzymes, their regulation across bacteria in the presence of keratin varies. Surprisingly, *F. gondwanense* DSM13020^T^ showed a higher number of upregulated peptidases and oxidoreductases than keratinolytic organisms, indicating that fervidobacteria may not make use of a large number of these enzymes for effective feather breakdown. The number of overrepresented peptidases identified in the transcriptomics and proteomics analyses was similar in the three studied strains. However, the number of overexpressed oxidoreductases, among the total number of these enzymes identified in the proteomes, was higher in the transcriptomics study, particularly in the early log phase, consistent with the hypothesis that oxidoreductases participate in the first steps of keratin degradation. Although many proteases have shown keratinolytic activity relying on the previous action of oxidoreductases, only a few can at least partially degrade keratin without accessory agents and have thus been categorized as “true keratinases” (Qiu et al., 2020). Two S8 proteases, one from *Bacillus* sp. AH-101 (Takami et al., 1990) and another from the fungus *Onygena corvina* (Huang et al., 2015), met these criteria. *F. pennivorans* T has three homologues of these keratinases: QIV78926.1, QIV78374.1 and QIV78937.1, respectively. QIV78374.1 was upregulated with a fold change of 7.68 in the proteomics analysis, and QIV78937.1 was overexpressed in both the transcriptomics and proteomics analyses, with fold changes of 1.76 and 3.08, respectively. Therefore, these three proteases are potential candidates for catalogs of true keratinases. While sources and mechanisms of action of keratinases are variate, with a wide distribution both in prokaryotic and eukaryotic organisms (Qiu et al., 2020), these three proteases can be classified as S8 serine endoproteases, a group of enzymes known to be involved in keratin degradation (Huang et al., 2015). An identical ortholog of QIV78937.1, termed fervidolysin (PDB 1R6V), has been described and expressed (Kluskens et al., 2002). Its 1.7 Å crystal structure showed four different domains, two sandwich domains, a 14 kDa propetide and a catalytic triad Asp_41_-His_79_-Ser_260_, composing a 58 kDa mature protein (Kim et al., 2004). An ortholog of the thermostable alkaline protease from *Bacillus* sp. AH-101was also identified in the upregulated proteome of *F. pennivorans* T (QIV78926.1). All these proteases are thermostable and active at high pH (10–12) (Qiu et al., 2020).

Furthermore, six peptidases which were overrepresented in the feather cultures of *F. pennivorans* T, *Fervidobacterium* sp. GSH and/or *F. islandicum* H-21^T^ were also confirmed to be overexpressed in the presence of keratin in a previous work (Kang et al., 2020) on *F. islandicum* AW-1: QIV78895.1, QIV78374.1, QIV78699.1, QIV79319.1, QIV79051.1 and QIV79147.1. Current work is progressing on enzymatic assays including these and other proteases, showing promising results. Proteomic and transcriptomic results often show a low correlation (Gygi et al., 1999), especially in bacteria. Thus, it is challenging to combine the data from both approaches (Nie et al., 2006). Here, the STRING networks that merged the upregulated proteins and overexpressed genes showed robust and meaningful connections across the overrepresented features, suggesting that most of them were linked to the response of these organisms to the presence of feather keratin in the environment. However, it is worth noting that non-overrepresented features may also participate in the degradation process, as well as others whose current annotation is not accurately solved.

## Conclusion

Based on the keratinolytic assessment of the 11 available strains of the genus *Fervidobacterium*, 6 strains, namely *F. changbaicum* CBS-1^T^, *F. islandicum* H-21^T^, *F. pennivorans T*, *F. pennivorans* DSM9078^T^, *Fervidobacterium* sp. GSH and *Fervidobacterium* sp. 21710 showed clear activity, completely degrading chicken breast feathers within 72 h at high temperatures (65–80°C). *F. islandicum* H-21^T^, *F. pennivorans* T, and *Fervidobacterium* sp. GSH were the most active organisms, with *F. pennivorans* disintegrating chicken feathers after 48 h at 70°C. The proteomics results revealed that only a small fraction of the proteome in the active strains responded to this condition and was upregulated in the presence of feathers, suggesting that these bacteria do not need major enzymatic machinery to break down feather keratin. A higher number of reductase-encoding genes were found in the early log phase of the overexpressed transcriptomes, implying the involvement of these enzymes in the initial stages of keratin degradation, congruent with the current keratin degradation hypothesis. Furthermore, three potential “true keratinases” were identified in *F. pennivorans* T: QIV78374.1, QIV78926.1, and QIV78937.1 but were downregulated in the feather cultures of the non-keratinolytic *F. gondwanense* DSM13020^T^. Homologs of these enzymes have already been cataloged as true keratinases, which are active even in the absence of helper oxidoreductases. The enzymes described here could expand the current catalog of available keratinolytic enzymes and may be integrated into industrial applications to help to mitigate the ecological and environmental challenges associated with feather waste accumulation.

## Data Availability

Mass spectrometry proteomics data are available from the ProteomeXchange Consortium via the PRIDE partner repository under the dataset identifiers PXD054267 and 10.6019/PXD054267 (*Fervidobacterium pennivorans* T), PXD054278, and 10.6019/PXD054278 (*Fervidobacterium* sp. GSH), PXD054282 and 10.6019/PXD054282 (*Fervidobacterium pennivorans* DSM 9078T), PXD054272 and 10.6019/PXD054272 (*Fervidobacterium islandicum* H-21T), and PXD054280 and 10.6019/PXD054280 (*Fervidobacterium* sp. DSM 21710), PXD054285 and 10.6019/PXD054285 (*Fervidobacterium changbaicum* CBS-1T), and PXD054269 and 10.6019/PXD054269 (*Fervidobacterium gondwanense* DSM13020T). The equivalence between the locus tag codes in the PRIDE tables and the accession numbers available in GenBank database is available in Supplementary Table S7.

## Footnotes

^1^ https://www.dsmz.de

^2^ https://jcm.brc.riken.jp

^3^ https://github.com/ncbi/pgap

^4^ https://eurofinsgenomics.eu/

^5^ https://digitalinsights.qiagen.com/

^6^ https://CRAN.R-project.org/package=pheatmap
