# Supplementary material for: Transcriptomic and proteomic insights into feather keratin degradation by Fervidobacterium
Source: Front Microbiol. 2025 Apr 16;16:1509937. doi: 10.3389/fmicb.2025.1509937 (PMC12042847; doi:10.3389/fmicb.2025.1509937)
Supplement: Supplementary file 1 [file Data_Sheet_1.zip › SUPPLEMENTARY_MATERIAL.docx]

***Supplementary Material***


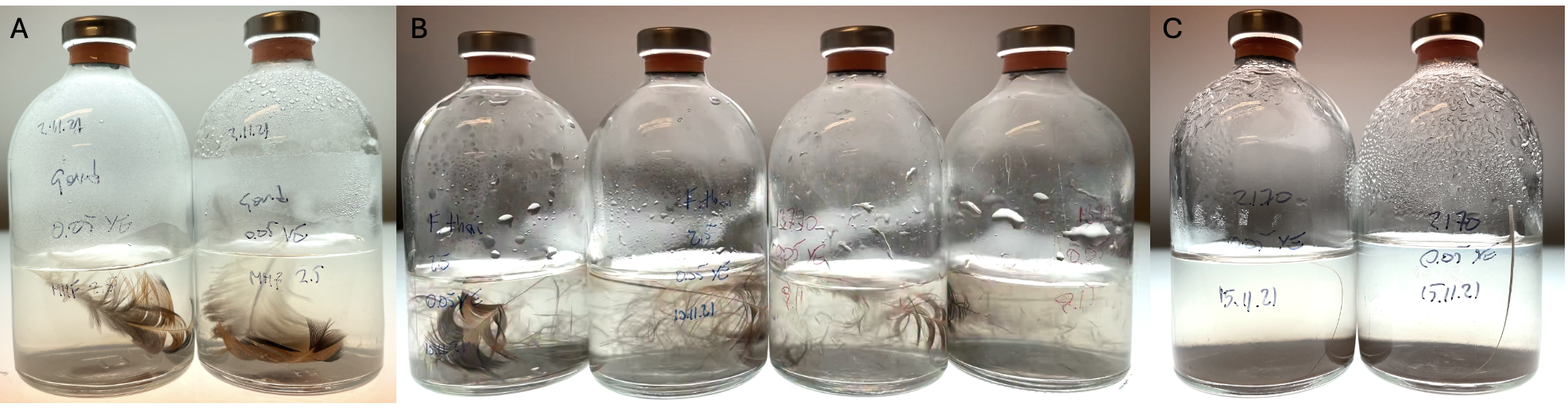


Figure S1. Feather cultures after 72 hours incubation at the optimum temperature of each organism. The panel illustrates an example of negative degradation by *F. gondwanense* DSM 13020^T^ (A), partial degradation (B) by *Fervidobacterium thailandense* FC2004^T^ (left) and *Fervidobacterium* sp. 13770 (right), and positive feather degradation by *Fervidobacterium* sp. 21710 (C).

Table S1. Significantly upregulated reductases and peptidases identified in the proteomics analysis.

| Strain | Reductases | Peptidases |
| --- | --- | --- |
| *F. pennivorans* T | QIV79098.1, QIV78923.1, QIV78416.1, QIV77819.1, QIV77827.1, QIV77828.1, QIV79107.1, QIV77984.1, QIV78156.1, QIV79321.1, QIV79104.1 | QIV78374.1, QIV78937.1, QIV78118.1, QIV78721.1, QIV78128.1, QIV78519.1, QIV78659.1, QIV78935.1 |
| *F*. sp. GSH | XEY12508.1, XEY12574.1, XEY12575.1, XEY12619.1, XEY12748.1, XEY12876.1, XEY12909.1, XEY13006.1, XEY13044.1, XEY13131.1, XEY11452.1, XEY11467.1, XEY11615.1, XEY11670.1, XEY12233.1 | XEY12978.1, XEY13048.1, XEY11555.1, XEY11654.1, XEY11656.1, XEY11827.1, XEY11881.1, XEY11904.1, XEY13256.1, XEY12292.1 |
| *F. islandicum* H-21^T^ | XEY10670.1, XEY10680.1, XEY10959.1, XEY11028.1, XEY11294.1, XEY09412.1, XEY09642.1, XEY09931.1, XEY10235.1, XEY10273.1, XEY10278.1 | XEY09446.1, XEY09564.1, XEY10359.1 |
| *F. pennivorans* DSM9078^T^ | AFG35434.1, AFG36043.1, AFG35855.1, AFG35907.1, AFG35218.1, AFG34372.1, AFG34623.1, AFG34767.1, AFG34482.1, AFG35121.1 | AFG35263.1, AFG36110.1, AFG35535.1, AFG35419.1, AFG35693.1, AFG35985.1, AFG35421.1, AFG35422.1, AFG35595.1, AFG35315.1, AFG34899.1 |
| *F*. sp. 21710 | XEY08434.1, XEY08439.1  XEY08680.1, XEY08776.1  XEY09050.1, XEY09253.1  XEY09254.1, XEY09276.1  XEY07453.1, XEY07596.1  XEY07635.1, XEY07638.1  XEY07759.1, XEY08049.1 | XEY08373.1, XEY09403.1  XEY08773.1, XEY08893.1  XEY09063.1, XEY09064.1  XEY09065.1, XEY09211.1  XEY07591.1 |
| *F. changbaicum* CBS-1^T^ | QAV33587.1, QAV32804.1, QAV32859.1, QAV32610.1, QAV32803.1, QAV32460.1, QAV33032.1, QAV32818.1, QAV33975.1, QAV33648.1, QAV32817.1, QAV32459.1, QAV33259.1, QAV32468.1, QAV33453.1, QAV33912.1, QAV34178.1, QAV34135.1, QAV32461.1, QAV33610.1 | QAV33664.1, QAV33237.1, QAV32663.1, QAV33017.1, QAV33019.1, QAV32689.1, QAV33020.1 |
| *F. gondwanense* DSM13020^T^ | XEY05451.1, XEY04463.1, XEY04488.1, XEY04493.1, XEY04527.1, XEY04697.1, XEY04765.1, XEY04978.1, XEY05070.1, XEY05113.1, XEY05240.1, XEY05241.1, XEY05277.1, XEY05301.1, XEY03538.1, XEY03741.1, XEY03804.1, XEY03835.1, XEY03884.1, XEY04030.1, XEY04066.1, XEY05430.1, XEY04222.1, XEY04225.1, XEY04320.1 | XEY04610.1, XEY04672.1, XEY04683.1, XEY04784.1  XEY05389.1, XEY05042.1  XEY05063.1, XEY05364.1  XEY03519.1, XEY03572.1  XEY03689.1, XEY03860.1  XEY03900.1, XEY04049.1  XEY04186.1, XEY04199.1 |


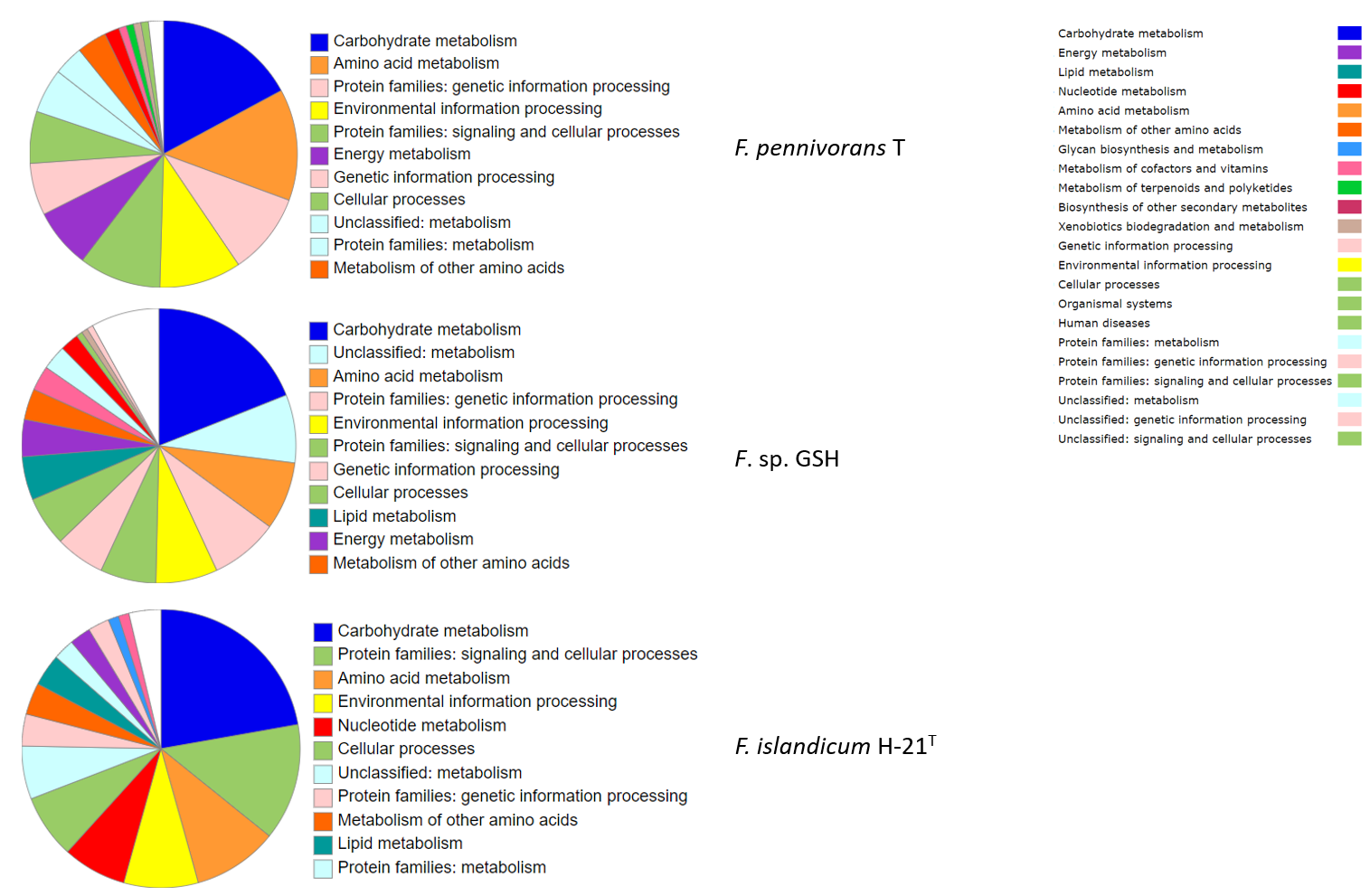


Figure S2. Pie chart plots illustrating an overview of the global metabolism of *F. pennivorans* T, *Fervidobacterium* sp. GSH and *F. islandicum* H-21^T^ based on the KEGG annotation of the upregulated proteome of these strains.

Table S2. General characteristics of the transcriptome of *F. pennivorans* T, after trimming and mapping to the reference genome.

| Substrate | Incubation (h) | Read count | Paired, mapped pairs (%) | Paired, broken pairs (%) | Paired, not mapped (%) | Coverage |
| --- | --- | --- | --- | --- | --- | --- |
| Glucose | 18 | 43,964,348 | 93.62 | 1.72 | 4.67 | 3,315 |
| Glucose | 18 | 36,959,162 | 92.56 | 1.96 | 5.49 | 2,787 |
| Glucose | 18 | 39,774,932 | 91.74 | 1.91 | 6.35 | 2,999 |
| Feather | 18 | 32,434,882 | 94.4 | 1.63 | 3.97 | 2,446 |
| Feather | 18 | 37,240,114 | 94.36 | 1.64 | 4.01 | 2,808 |
| Feather | 18 | 28,525,848 | 94.2 | 1.67 | 4.13 | 2,151 |
| Feather | 40 | 36,503,984 | 92.56 | 1.88 | 5.56 | 2,753 |
| Feather | 40 | 31,470,658 | 93.45 | 1.89 | 4.66 | 2,373 |
| Feather | 40 | 48,896,222 | 94.57 | 1.60 | 3.83 | 3,687 |

Table S3. General characteristics of the transcriptome of *F.* sp. GSH, after trimming and mapping to the reference genome.

| Substrate | Incubation (h) | Read count | Paired, mapped pairs (%) | Paired, broken pairs (%) | Paired, not mapped (%) | Coverage |
| --- | --- | --- | --- | --- | --- | --- |
| Glucose | 18 | 33,020,142 | 92.6 | 2.06 | 5.33 | 2,465 |
| Glucose | 18 | 37,183,752 | 94.1 | 1.76 | 4.14 | 2,775 |
| Glucose | 18 | 31,585,874 | 93.65 | 2.11 | 4.24 | 2,358 |
| Feather | 18 | 33,626,760 | 95.17 | 1.55 | 3.28 | 2,510 |
| Feather | 18 | 22,966,682 | 94.29 | 1.8 | 3.92 | 1,714 |
| Feather | 18 | 33,924,354 | 95.03 | 1.67 | 3.3 | 2,532 |
| Feather | 40 | 24,629,448 | 94.29 | 1.85 | 3.86 | 1,838 |
| Feather | 40 | 37,445,318 | 94.38 | 1.69 | 3.93 | 2,795 |
| Feather | 40 | 30,350,910 | 94.52 | 1.66 | 3.82 | 2,265 |

Table S4. General characteristics of the transcriptome of *F. islandicum* H-21^T^, after trimming and mapping to the reference genome.

| Substrate | Incubation (h) | Read count | Paired mapped pairs (%) | Paired broken pairs (%) | Paired not mapped (%) | Coverage |
| --- | --- | --- | --- | --- | --- | --- |
| Glucose | 18 | 36,085,414 | 93.32 | 1.24 | 5.44 | 2,485 |
| Glucose | 18 | 31,478,470 | 92.58 | 1.37 | 6.05 | 2,168 |
| Glucose | 18 | 33,180,524 | 91.51 | 1.19 | 7.31 | 2,285 |
| Feather | 18 | 28,911,624 | 94.1 | 1.22 | 4.68 | 1,991 |
| Feather | 18 | 35,714,352 | 94.48 | 1.14 | 4.37 | 2,460 |
| Feather | 18 | 24,887,512 | 88.5 | 1.13 | 10.37 | 1,714 |
| Feather | 40 | 31,638,814 | 86.95 | 1.1 | 11.96 | 2,179 |
| Feather | 40 | 30,665,446 | 92.26 | 1.16 | 6.58 | 2,112 |
| Feather | 40 | 27,256,484 | 91.83 | 1.27 | 6.9 | 1,877 |
